# Comparative Verification of the Accuracy of Implant Models Made of PLA, Resin, and Silicone

**DOI:** 10.3390/ma16093307

**Published:** 2023-04-23

**Authors:** Kana Wakamori, Koudai Nagata, Toshifumi Nakashizu, Hayato Tsuruoka, Mihoko Atsumi, Hiromasa Kawana

**Affiliations:** 1 Department of Oral and Maxillofacial Implantology, Kanagawa Dental University, 82 Inaoka-cho, Yokosuka 238-8580, Japan; 2 Division of the Dental Practice Support, Kanagawa Dental University, 82 Inaoka-cho, Yokosuka 238-8580, Japan

**Keywords:** 3D printing, fused filament fabrication (FFF), digital light processing (DLP), polylactic acid (PLA), dental implant

## Abstract

Polylactic acid (PLA) has gained considerable attention as an alternative to petroleum-based materials due to environmental concerns. We fabricated implant models with fused filament fabrication (FFF) 3D printers using PLA, and the accuracies of these PLA models were compared with those of plaster models made from silicone impressions and resin models made with digital light processing (DLP). A base model was obtained from an impact-training model. The scan body was mounted on the plaster, resin, and PLA models obtained from the base model, and the obtained information was converted to stereolithography (STL) data by the 3D scanner. The base model was then used as a reference, and its data were superimposed onto the STL data of each model using Geomagic control. The horizontal and vertical accuracies of PLA models, as calculated using the Tukey–Kramer method, were 97.2 ± 48.4 and 115.5 ± 15.1 μm, respectively, which suggests that the PLA model is the least accurate among the three models. In both cases, significant differences were found between PLA and gypsum and between the PLA and resin models. However, considering that the misfit of screw-retained implant frames should be ≤150 µm, PLA can be effectively used for fabricating implant models.

## 1. Introduction

The history of dental implants in current use can be traced back to the first clinical use of root-shaped titanium implants in 1965, which are still in use today. The bonding mode between bone and titanium is called osseointegration [1]. Various surface treatments, including blasting, etching, sandblasting, and anodizing, are used to ensure osseointegration [2,3,4]. Oates et al. reported that implant stability can be accelerated by two weeks if implants are sandblasted and surface-treated with acid etching [5]. In a 20-year follow-up study of 631 patients and 1472 implant bodies, Cheng et al. reported a 94% implant survival rate [6]. Various surface treatment techniques have accelerated implant stability and established the long-term prognosis of implant therapy [7]. In recent years, digital technology has been widely used in implant treatment. The concepts of top-down treatment and static and dynamic navigation in surgery have become widespread, allowing for safe and esthetic implant treatment for patients [8,9,10]. In implant prosthetic treatment, intraoral scanners (IOSs) and computer-aided design/computer-aided manufacturing (CAD/CAM) have been applied to single-tooth and multiple-tooth defect cases. This has enabled the digitization of almost all processes, leading to improved accuracy of prosthetics, shorter treatment times, and reduced fabrication times of technical work [11,12,13].

The Sustainable Development Goals, adopted at the 2015 United Nations Summit, are currently attracting attention from the environmental perspective. In particular, Goal 12, “ensure sustainable patterns of consumption and production”, targets waste reduction [14]. According to the WHO, biomedical waste (BMW) is one of the most important categories of waste, posing significant potential risks to people and the environment. BMW is defined as “the generation of waste in medical institutions, medical research facilities, laboratories, and private practices”. The global growth of the medical and dental sectors and the increase in disposable products have resulted in the generation of large amounts of medical and dental waste [15]. A survey on dental waste in Greece [16] reported that 141 kg of waste was collected from 20 dental clinics with a total patient population of 2542, with 8% of the total weight being household waste and 92% hazardous waste. Koolivand et al. also reported that in Urmia, Iran, general dental offices accounted for 58.94 kg of waste per day, specialized dental clinics for 17.92 kg/day, dental clinics for 10.22 kg/day, household waste for 35.46%, potentially infectious waste for 32.24%, and toxic waste for 11.83%, while chemical and pharmaceutical wastes accounted for 5.56% of the total [17]. According to the Survey Report on Industrial Waste Discharge and Disposal by Sector reported by Japan’s Ministry of the Environment in 2021, the amount of industrial waste discharged by the medical industry accounted for 438 (thousand tons) in 2021. Our dental hospital in Japan also generates approximately 2660 kg of industrial waste per year. Therefore, the reduction, management, and reuse of dental waste is a challenge that healthcare professionals face [18]. Papi et al. discussed how impression materials and plaster casts with blood or saliva on them can be a source of infection [19]. Frahdian et al. stated that alginate impressions are one of the reasons for the increase in dental waste [20]. Silicone impression material, alginate, and plaster are considered industrial waste in the field of dentistry in Japan. Plaster is commonly used to fabricate dental models, but plaster models are gradually being replaced by resin models sculpted by light-based 3D printers due to the widespread use of IOSs and 3D printers in dentistry [21,22]. This is because the light-based 3D printer method is considered to have better accuracy. Ishida et al. [23] fabricated dental patterns and verified their accuracy using consumer 3D printers such as a fused filament fabrication (FFF) device, a stereolithography (SLA) device, and two types of dental 3D printers (a Multijet device and an SLA device). As a result, the surface roughness of the civilian consumer FFF devices is significantly larger than that of the SLA devices, and the accuracy of the SLA devices is better than that of the civilian FFF devices. Kim et al. [24] measured the accuracy of models fabricated using SLA, digital light processing (DLP), FFF, and PolyJet. Overall tooth measurements were 88 ± 14 μm for SLA, 76 ± 14 μm for DLP, 99 ± 14 μm for FFF, and 68 ± 9 μm for PolyJet, indicating that 3D printing technology is applicable to dental models. SLA uses a UV laser to form the liquid resin. DLP uses a projector to project an image of one layer onto the entire surface of the build platform and cures the entire layer on a “surface” rather than curing it at “dots” as with the SLA devices [25]. In dentistry, SLA and DLP are used to produce orthodontic devices and surgical guides for implant surgery due to their accuracy [26,27]. However, light-based 3D printers can only use resin, and light-mediated resin cannot be broken down since it is a polymer. Therefore, resin models cannot be reused and they do not help reduce industrial waste [28]. That is why we turned our attention to FFF. In the FFF manufacturing process, raw material is melted to form an object called a filament. This material is pulled by a drive wheel through filaments placed on a roll and heated by a temperature-controlled nozzle head to produce a semi-liquid material that is precisely extruded and guided layer by layer to produce the desired object [29]. FFF uses various filaments, such as polylactic acid (PLA), acrylonitrile butadiene styrene, and polyethylene terephthalate [30].

Since PLA is present in the filament used for FFF, we believe that PLA could be reused after the model is fabricated and remolded, thereby reducing industrial waste. PLA is characterized by decomposition into water and carbon dioxide under composting conditions of high temperature, high humidity, and the presence of microorganisms [31,32]. PLA is widely used in medical practice, and its biocompatibility has been widely reported [33,34,35]. However, there have been few reports on the use of FFF or PLA in dentistry. Benli et al. [36] and Molinero-Mourelle et al. [37] reported the superiority of PLA as a provisional crown. Crenn et al. [38] compared the mechanical properties of PLA to those of conventional resins and reported that PLA has mechanical properties similar to those of conventional resins with low porosity and could be used for provisional crowns. However, Park et al. [39] reported that three-unit provisional crowns fabricated from SLA and DLP had superior bending strength, and it was difficult to fabricate three-unit provisional crowns from FFF. Results may vary depending on the nature of the 3D printer and the filament. The glass transition temperature of PLA is as low as 60 °C. Methods to increase the heat resistance of PLA have been studied, but no clear method has been found that does not impair the biodegradability of PLA [40,41]. Therefore, it is currently difficult to use PLA as a crown, and practically, it is more useful for making dental models.

Regarding FFF, Muta et al. [42] compared plaster models and polyvinyl alcohol (PVA) models made with FFF and reported the usefulness of FFF and PVA. Wang and Su [43] compared the accuracy of edentulous trays fabricated using DLP and FFF with that of conventional manual edentulous trays and found the digitally fabricated trays to have higher precision. Research on filament reuse has also been conducted. Lagazzo et al. [44] reported the effectiveness of PLA and poly (3-hydroxybutyrate-co-3-hydroxyvalerate)-based biocomposites for composite material recycling. Vidakis et al. also reported that PA12 polymers can be reused up to three times [45]. Anderson et al. [46] compared the mechanical properties of virgin PLA and one-time-recycled PLA and reported a 10.9% decrease in tensile strength, 6.8% increase in shear strength, and 2.4% decrease in the hardness of the reused filaments. While there are many reports on the reuse of PLA, no clear process for reuse has been defined. Majgaonkar et al. [47] believe that it is important to have a sustainable strategy for recycling PLA waste due to current environmental concerns, although recycling PLA will degrade its mechanical properties. They also considered recycling strategies that involve the alcoholysis of post-consumer PLA into lactic acid esters. We believe that the use of an IOS and PLA to fabricate and reuse implant models would lead to dental care with reduced waste [48]. However, to the best of our knowledge, there are no reports documenting the accuracy of implant models using FFF or PLA. In our previous study, we compared dental models made of PLA with those made of resin and plaster, and reported that the PLA models were equally accurate [49]. Therefore, in this study, we aimed to compare the accuracy of implant plaster models made from silicon impression material and plaster, implant resin models made with DLP, and implant PLA models made with FDM.

## 2. Materials and Methods

**Figure 1:**
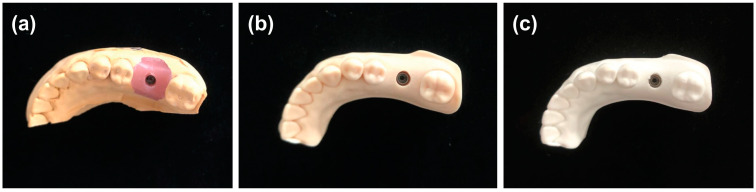
Different types of constructed models. (**a**) A silicone impression was made on the mother model, the lab analog was attached to the impression coping, and plaster was injected to make a plaster model. (**b**) Impressions were taken on the base model using Trios3^®^, and resin models were fabricated using cara Print 4.0 pro based on the obtained STL data. (**c**) Impressions were taken on the base model using Trios3^®^, and PLA models were fabricated using Moment M350 based on the obtained STL data. STL: stereolithography.

Straumann^®^ ∮4.1 × 10 implants (bone-level tapered implant, Basel, Switzerland) were placed on a jaw model for implant training ([D18D-KP.80]; NISSIN, Tokyo, Japan), Switzerland) and were used as the base model. Next, the scan body (S-WAVE, SHOFU INC, Tokyo, Japan) was mounted and scanned with a 3D scanner (Ceramill Map^®^ 400; Amann Girrbach, Vienna, Austria) to acquire stereolithography (STL) data of the mother model. The process of making each model and obtaining STL data is described below. The models are shown in Figure 1. This study was conducted in compliance with SQUIRE guidelines.

### 2.1. STL Data Acquisition for Plaster Implant Models

Precision impressions were made on base models with impression copings using silicone (Aquasil Ultra^®^; Dentsply Sirona, York, PA, USA). The lab analog was then mounted on an impression coping, and plaster (New Fujirock^®^; GC, Tokyo, Japan) was poured to fabricate the implant model. A scan body (S-WAVE, Shofu, Tokyo, Japan) was attached, scanned with a 3D scanner, and converted to STL data.

### 2.2. STL Date Acquisition for Resin Implant Models

Digital impressions were made by the IOS (Trios^®^ 3; 3shape, Copenhagen, Denmark) on the base model with the scan body attached, and then DLP (cara^®^ Print 4.0 pro; Kulzer Japan Co., Ltd., Tokyo, Japan) and light-curing resin (dima^®^ Print Stone; Kulzer Japan Co., Ltd., Tokyo, Japan) were used to fabricate the resin models. The layer thickness was 50 µm. The resin model was fitted with a scan body and converted to STL data using a 3D scanner.

### 2.3. STL Data Acquisition for PLA Implant Models

Digital impressions of the base model were taken with the scan body attached; FFF (Moment^®^ M350; Moment Co., Ltd., Seoul, Republic of Korea) and a 1.75 mm PLA filament from Moment^®^ (Moment Co., Ltd., Seoul, Republic of Korea) were used to fabricate the PLA models. The fabrication conditions were as follows: modeling temperature of the material = 230 °C and layer thickness = 100 µm. The PLA model was fitted with a scan body and converted to STL data using a 3D scanner. No surface polishing or chemical treatment was performed after printing. The specifications of the 3D printers are shown in Table 1.

### 2.4. Measurement of Accuracy

**Figure 2:**
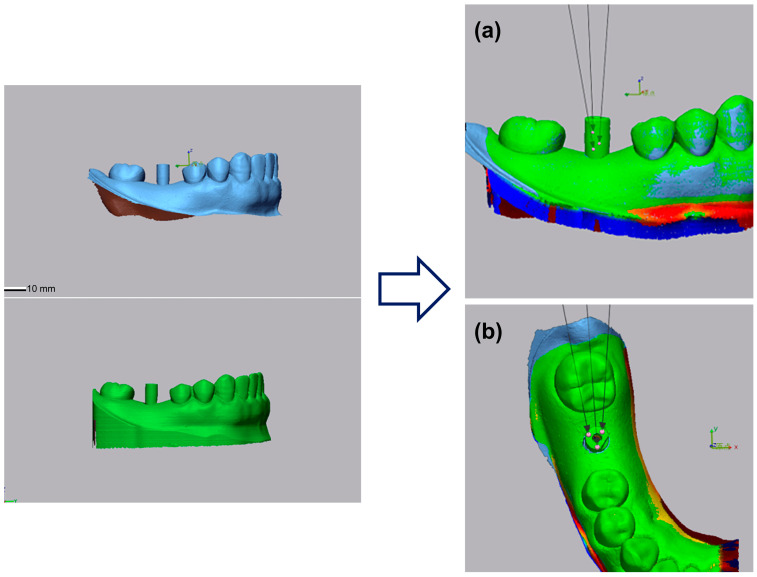
Stereolithography (STL) of the base model superimposed on each of the models to measure accuracy. For horizontal and vertical accuracies, three points were randomly selected, and their average was used as the result. (**a**) Horizontal and (**b**) vertical accuracy.

After obtaining the STL data for each model, the accuracy of the scanned body of plaster, resin, and PLA models was measured using Geomagic^®^ Control (3D Systems, Washington, DC, USA) based on the STL data of the base model. The superimposing of STL data was performed after trimming the excess data, followed by manual alignment based on three landmarks, and best-fit registration was used for greater accuracy. The average of the results was obtained by randomly selecting three points from the superimposed scan body data (Figure 2). The scan bodies were all in the same orientation. Five models were designed for each. Accuracy was measured in two directions, viz. horizontal and vertical.

### 2.5. Statistical Analysis

The accuracy of the model was verified through the Tukey–Kramer method using a bell curve in Excel (Social Survey Research Information Co., Ltd., Tokyo, Japan). Continuous data are expressed as mean ± standard deviation. Differences with a *p* value < 0.05 were considered statistically significant.

## 3. Results and Discussion

**Figure 3:**
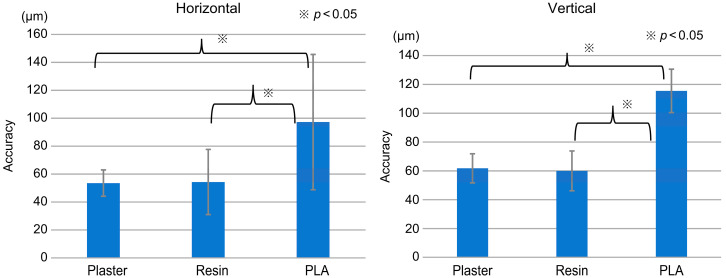
Comparison of the horizontal and vertical accuracies of the three models. We can observe significant differences in both horizontal and vertical accuracies between plaster and polylactic acid (PLA) and resin and PLA.

In this study, horizontal accuracies of 53.4 ± 9.4, 54.3 ± 23.4, and 97.2 ± 48.4 μm were obtained for plaster, resin, and PLA, respectively (*p* < 0.05), while the corresponding vertical accuracies were 61.8 ± 10.1, 60 ± 13.8, and 115.5 ± 15.1 μm (*p* < 0.001). In both cases, PLA had the lowest accuracy. Significant differences in horizontal accuracies were found between PLA and plaster and PLA and resin. Vertical accuracies were similarly significantly different between PLA and resin (Figure 3).

The findings of this study demonstrate that the accuracy of PLA models was inferior to that of the resin and plaster models both vertically and horizontally. However, with the improved accuracy of 3D printers, PLA could be used as a new material in dentistry. Furthermore, due to the advantages of its characteristics, PLA can be reused to reduce industrial waste and carbon dioxide emissions [50].

Reports on model-less prosthetic fittings are scarce; a systematic review by Joda et al. found only two reports on model-less crown fits and one on implant superstructure fit [51]. Joda et al. also compared the accuracy of 10 superstructures fabricated without models and 10 superstructures with models and reported that the model-less superstructures required less adjustment and time to fit [52]. However, Mühlemann et al. reported that the fabrication of fittings with digital models should be considered as these were more accurate than those made with conventional plaster models [53].

When fabricating a superstructure, a model is necessary for creating the bond between the zirconia and the titanium base. Particularly in the case of single-tooth implants, the titanium base has an anti-rotation mechanism, and if a model is not used, minor misalignments may prevent the implant from fitting in the mouth. Therefore, the accuracy of the model is important. Geomagic^®^ Control, which was used to measure the accuracy in this study, is widely used in dentistry to verify the crown fit and IOS accuracy by comparing STL data [54,55,56].

Hanon et al. fabricated cylindrical specimens using FFF and reported that the modeling accuracy was as high as 98.56–99.64% [57]. The results of this study show that the accuracy of the PLA model was lower than that of the other models. We hypothesize that the accuracy loss of the PLA model was due to the difference in layer thickness between the two 3D printers. However, Kamio et al. [58] reported that the layer thickness of the FFF did not cause a significant decrease in accuracy. Additionally, FFF is limited in its ability to model detailed areas [59]. George et al. [60] reported that models fabricated with FFF are susceptible to shrinkage and warping deformation during the cooling process of the thermoplastic resin, and that geometric inaccuracies occur when models of vertebral bodies and other spinous processes are fabricated. When an implant model is fabricated using a 3D printer, a lab analog corresponding to the implant is inserted from the basal surface after the modeling. In contrast to the smooth insertion of the resin and plaster models, the PLA models could not be inserted without grinding with a laboratory bur, which could be the reason for the lower accuracy of the PLA models. It is necessary to verify whether the accuracy of dental models can be improved by using more accurate FFF or changing the modeling direction [61,62].

With respect to the fit of screw-retained implant frames, Katsoulis et al. used scanning electron microscopy to evaluate the micro-gap between the screw-retained zirconia frame and the implant using the one-screw test [63]. They reported that the micro-gap of the cast cobalt chrome frame was 236 µm, while that of the zirconia frame was 18 µm, and an acceptable distortion of <50–120 µm was noted. Al-Meraikhi et al. [64] measured the fit of the implant to the zirconia frame using an industrial computed tomography (CT) scanner and volume graphics analysis software and found that the fit was 93.8 ± 30 µm. The passive fit was reported to be acceptable at 135 µm. Yilmaz et al. [65] also reported that the marginal discrepancy of screw-fixed titanium and zirconia frameworks and abutments were 102 µm and 94 µm, respectively, measured using an industrial CT scanner and 3D volume software; they also reported clinically acceptable misfit values ranging from 10 µm to 150 µm. Many other researchers have reported misfit limits of < 150 µm for the precision of fit of screw-retained implants [66,67,68].

With respect to the accuracy of scan bodies using Geomagic^®^ control, Mühlemann et al. [53] measured the accuracy of impressions using IOSs in five patients with a single missing tooth and teeth on both sides of the edentulous space. Three consecutive impressions were taken with each IOS to measure for accuracy. They reported that the scan body misfit was 57.2 ± 32.6 and 88.6 ± 46 µm for the iTero and Trios systems, respectively. Gedrimiene et al. [69] used conventional silicone-based impressions and IOS-based digital impressions and reported that the scan body misfit was 70.8 ± 59 µm. The horizontal and vertical accuracies of PLA models in our study were 97.2 ± 48.4 μm and 115.5 ± 15.1 μm, respectively. The accuracy of PLA models was found to be lower than that of the resin and plaster models. However, resin and plaster models cannot be reused and eventually become industrial waste. Since the misfit of the screw-fixed implant frame was <150 µm, PLA models can be used as implant models. Reusing PLA models may lead to a reduction in industrial waste and carbon dioxide emissions. The accuracy can be further improved by using a verification jig [70]. Although its accuracy needs to be verified in clinical practice, we believe that in the future, the use of PLA can contribute to reducing dental waste.

Three-dimensional printers are gaining popularity in medicine, but there are some concerns. SLA and DLP produce toxic substances and odors during the production process [71]. PLA is known to release volatile organic compounds (VOCs) during printing as well. Chan et al. [72] measured the concentration of VOCs in one printer and in a printing room when three printers were operating simultaneously. Total VOC concentrations were reported, with isopropyl alcohol being the primary VOC and both being below occupational exposure limits. Wojtyła et al. [73] reported that during PLA molding, methyl methacrylate was detected as a compound, accounting for 44% of total VOC emissions. Thus, ventilation and protection of the printing room are important because the emission of hazardous substances has been confirmed, even if within acceptable limits, during FFF modeling [74]. There is also much debate regarding the sterilization methods for PLA. Currently, the three most common industrially used sterilization methods for medical devices are ethylene oxide, gamma irradiation, and steam sterilization, which can significantly alter the properties of PLA. PLA cannot withstand steam sterilization due to its low heat resistance. Gamma irradiation and ethylene oxide have lower sterilization temperatures and can be applied to heat-sensitive materials such as PLA. However, gamma irradiation degrades polymers, and ethylene oxide is toxic, carcinogenic, and allergenic, among other drawbacks [75,76,77]. There are reports that FFF is self-sterilizing due to the high-temperature, high-pressure extrusion process [78]. Davila et al. [79] report that there is no specific technology that can be applied to all materials used in biomedical devices and that new processes are needed to avoid these problems. They also report that hydrogen peroxide gas plasma and supercritical carbon dioxide are effective sterilization methods.

A limitation of this study is that only one type of FFF was used. We believe that further detailed and extensive studies can help in better comparing the accuracy of multiple FDMs in the future. It is also important to measure how much industrial waste can be reduced by using PLA for dental treatment, compared to other materials.

## 4. Conclusions

We hypothesized that the characteristics of PLA could be exploited to reuse it and reduce industrial waste in dentistry. This study compared the accuracy of implant models made of PLA with that of models made of plaster and resin. The PLA models were less accurate than the other two models but considering that the misfit of the screw-fixed superstructure was <150 µm, it could be used as a new material.

However, no clear method has been established regarding the reuse of PLA. Additionally, due to the mechanical properties of PLA, an exact sterilization method has not been determined. Solving these problems will make PLA reuse a reality.

## Figures and Tables

**Table 1:** Specifications of the 3D printers used in this study.

3D Printing Technique	3D Printer Used	Specifications
DLP ^†^	cara Print 4.0 pro (Kulzer Japan Co., Ltd., Tokyo, Japan)	Pixel size: XY 65.0 μm Laminating pitch: 30–150 μm Modeling size: 127 mm × 70 mm × 130 mm
FFF ^‡^	Moment M350 (Moment Co., Ltd., Seoul, Republic of Korea)	XYZ accuracy: XY 12 μm, Z 0.625 μm Laminating pitch: 0.05–0.3 mm Modeling size: 350 mm × 190 mm × 196 mm Nozzle: 0.4 mm

Resin models were constructed using cara Print 4.0 pro, and PLA models were constructed using Moment M350. ^†^ Digital light processing, ^‡^ fused filament fabrication.

## Data Availability

The datasets used and/or analyzed during the current study are available from the corresponding author upon reasonable request.

## References

[1] Abraham C.M. (2014). A Brief Historical Perspective on Dental Implants, Their Surface Coatings and Treatments. Open Dent. J..

[2] Glauser R., Ree A., Lundgren A., Gottlow J., Hammerle C.H., Scharer P. (2001). Immediate Occlusal Loading of Brånemark Implants Applied in Various Jawbone Regions: A Prospective, 1-Year Clinical Study. Clin. Implant. Dent. Relat. Res..

[3] Bornstein M.M., Schmid B., Belser U.C., Lussi A., Buser D. (2005). Early loading of non-submerged titanium implants with a sandblasted and acid-etched surface. 5-year results of a prospective study in partially edentulous patients. Clin. Oral Implant. Res..

[4] Bergkvist G., Koh K.-J., Sahlholm S., Klintström E., Lindh C. (2010). Bone density at implant sites and its relationship to assessment of bone quality and treatment outcome. Int. J. Oral Maxillofac. Implant..

[5] Oates T.W., Valderrama P., Bischof M., Nedir R., Jones A., Simpson J., Toutenburg H., Cochran D.L. (2007). Enhanced implant stability with a chemically modified SLA surface: A randomized pilot study. Int. J. Oral Maxillofac. Implant..

[6] Cheng Y.-C., Ewers R., Morgan K., Hirayama M., Murcko L., Morgan J., Bergamo E.T.P., Bonfante E.A. (2022). Antiresorptive therapy and dental implant survival: An up to 20-year retrospective cohort study in women. Clin. Oral Investig..

[7] Andrade C.A.S., Paz J.L.C., de Melo G.S., Mahrouseh N., Januário A.L., Capeletti L.R. (2022). Survival rate and peri-implant evaluation of immediately loaded dental implants in individuals with type 2 diabetes mellitus: A systematic review and meta-analysis. Clin. Oral Investig..

[8] Chackartchi T., Romanos G.E., Parkanyi L., Schwarz F., Sculean A. (2022). Reducing errors in guided implant surgery to optimize treatment outcomes. Periodontology 2000.

[9] Derksen W., Wismeijer D., Flügge T., Hassan B., Tahmaseb A. (2019). The accuracy of computer-guided implant surgery with tooth-supported, digitally designed drill guides based on CBCT and intraoral scanning. A prospective cohort study. Clin. Oral Implant. Res..

[10] Wang X., Shaheen E., Shujaat S., Meeus J., Legrand P., Lahoud P., Gerhardt M.D.N., Politis C., Jacobs R. (2022). Influence of experience on dental implant placement: An in vitro comparison of freehand, static guided and dynamic navigation approaches. Int. J. Implant. Dent..

[11] Russo L.L., Caradonna G., Biancardino M., De Lillo A., Troiano G., Guida L. (2019). Digital versus conventional workflow for the fabrication of multiunit fixed prostheses: A systematic review and meta-analysis of vertical marginal fit in controlled in vitro studies. J. Prosthet. Dent..

[12] Aktas G., Özcan N., Aydin D.H., Şahin E., Akça K. (2014). Effect of digitizing techniques on the fit of implant-retained crowns with different antirotational abutment features. J. Prosthet. Dent..

[13] Nagata K., Fuchigami K., Okuhama Y., Wakamori K., Tsuruoka H., Nakashizu T., Hoshi N., Atsumi M., Kimoto K., Kawana H. (2021). Comparison of digital and silicone impressions for single-tooth implants and two- and three-unit implants for a free-end edentulous saddle. BMC Oral Health.

[14] Valenzuela-Levi N. (2021). Poor performance in municipal recycling: The case of Chile. Waste Manag..

[15] Antoniadou M., Varzakas T., Tzoutzas I. (2021). Circular Economy in Conjunction with Treatment Methodologies in the Biomedical and Dental Waste Sectors. Circ. Econ. Sustain..

[16] Mandalidis A., Topalidis A., Voudrias E.A., Iosifidis N. (2018). Composition, production rate and characterization of Greek dental solid waste. Waste Manag..

[17] Koolivand A., Gholami-Borujeni F., Nourmoradi H. (2015). Investigation on the characteristics and management of dental waste in Urmia, Iran. J. Mater. Cycles Waste Manag..

[18] Sabbahi D.A., El-Naggar H.M., Zahran M.H. (2020). Management of dental waste in dental offices and clinics in Jeddah, Saudi Arabia. J. Air Waste Manag. Assoc..

[19] Papi P., Di Murro B., Penna D., Pompa G. (2020). Digital prosthetic workflow during COVID-19 pandemic to limit infection risk in dental practice. Oral Dis..

[20] Frahdian T., Hasratiningsih Z., Karlina E., Herdiyantoro D., Takarini V. (2018). Dental alginate impression waste as additional fertiliser for plant yields and soil quality. Padjadjaran J. Dent..

[21] Alharbi N., Osman R., Wismeijer D. (2016). Effects of build direction on the mechanical properties of 3D-printed complete coverage interim dental restorations. J. Prosthet. Dent..

[22] Srinivasan M., Kalberer N., Kamnoedboon P., Mekki M., Durual S., Özcan M., Müller F. (2021). CAD-CAM complete denture resins: An evaluation of biocompatibility, mechanical properties, and surface characteristics. J. Dent..

[23] Ishida Y., Miura D., Miyasaka T., Shinya A. (2020). Dimensional Accuracy of Dental Casting Patterns Fabricated Using Consumer 3D Printers. Polymers.

[24] Kim S.-Y., Shin Y.-S., Jung H.-D., Hwang C.-J., Baik H.-S., Cha J.-Y. (2018). Precision and trueness of dental models manufactured with different 3-dimensional printing techniques. Am. J. Orthod. Dentofac. Orthop..

[25] Borrello J., Nasser P., Iatridis J.C., Costa K.D. (2018). 3D printing a mechanically-tunable acrylate resin on a commercial DLP-SLA printer. Addit. Manuf..

[26] Maspero C., Tartaglia G.M. (2020). 3D Printing of Clear Orthodontic Aligners: Where We Are and Where We Are Going. Materials.

[27] Wegmüller L., Halbeisen F., Sharma N., Kühl S., Thieringer F.M. (2021). Consumer vs. High-End 3D Printers for Guided Implant Surgery—An In Vitro Accuracy Assessment Study of Different 3D Printing Technologies. J. Clin. Med..

[28] Hopewell J., Dvorak R., Kosior E. (2009). Plastics recycling: Challenges and opportunities. Philos. Trans. R. Soc. Lond. B Biol. Sci..

[29] Kristiawan R.B., Imaduddin F., Ariawan D., Arifin Z. (2021). A review on the fused deposition modeling (FDM) 3D printing: Filament processing, materials, and printing parameters. Open Eng..

[30] Micó-Vicent B., Perales E., Huraibat K., Martínez-Verdú F.M., Viqueira V. (2019). Maximization of FDM-3D-Objects Gonio-Appearance Effects Using PLA and ABS Filaments and Combining Several Printing Parameters: “A Case Study”. Materials.

[31] D’anna A., Arrigo R., Frache A. (2019). PLA/PHB Blends: Biocompatibilizer Effects. Polymers.

[32] Farah S., Anderson D.G., Langer R. (2016). Physical and mechanical properties of PLA, and their functions in widespread applications—A comprehensive review. Adv. Drug Deliv. Rev..

[33] Gai M., Li W., Frueh J., Sukhorukov G.B. (2018). Polylactic acid sealed polyelectrolyte complex microcontainers for controlled encapsulation and NIR-Laser based release of cargo. Colloids Surf. B Biointerfaces.

[34] Li Z., Wu T., Chen Y., Gao X., Ye J., Jin Y., Chen B. (2021). Oriented homo-epitaxial crystallization of polylactic acid displaying a biomimetic structure and improved blood compatibility. J. Biomed. Mater. Res. A.

[35] Ahuja R., Kumari N., Srivastava A., Bhati P., Vashisth P., Yadav P., Jacob T., Narang R., Bhatnagar N. (2020). Biocompatibility analysis of PLA based candidate materials for cardiovascular stents in a rat subcutaneous implant model. Acta Histochem..

[36] Benli M., Eker-Gümüş B., Kahraman Y., Huck O., Özcan M. (2021). Can polylactic acid be a CAD/CAM material for provisional crown restorations in terms of fit and fracture strength?. Dent. Mater. J..

[37] Molinero-Mourelle P., Canals S., Gómez-Polo M., Solá-Ruiz M., Highsmith J.D.R., Viñuela A. (2018). Polylactic Acid as a Material for Three-Dimensional Printing of Provisional Restorations. Int. J. Prosthodont..

[38] Crenn M.-J., Rohman G., Fromentin O., Benoit A. (2022). Polylactic acid as a biocompatible polymer for three-dimensional printing of interim prosthesis: Mechanical characterization. Dent. Mater. J..

[39] Park S.-M., Park J.-M., Kim S.-K., Heo S.-J., Koak J.-Y. (2020). Flexural Strength of 3D-Printing Resin Materials for Provisional Fixed Dental Prostheses. Materials.

[40] Koske D., Ehrmann A. (2021). Advanced Infill Designs for 3D Printed Shape-Memory Components. Micromachines.

[41] Yang Y., Xiong Z., Zhang L., Tang Z., Zhang R., Zhu J. (2016). Isosorbide dioctoate as a “green” plasticizer for poly(lactic acid). Mater. Des..

[42] Muta S., Ikeda M., Nikaido T., Sayed M., Sadr A., Suzuki T., Tagami J. (2020). Chairside fabrication of provisional crowns on FDM 3D-printed PVA model. J. Prosthodont. Res..

[43] Wang X., Su J. (2021). Evaluation of Precision of Custom Edentulous Trays Fabricated with 3D Printing Technologies. Int. J. Prosthodont..

[44] Lagazzo A., Moliner C., Bosio B., Botter R., Arato E. (2019). Evaluation of the Mechanical and Thermal Properties Decay of PHBV/Sisal and PLA/Sisal Biocomposites at Different Recycle Steps. Polymers.

[45] Vidakis N., Petousis M., Tzounis L., Maniadi A., Velidakis E., Mountakis N., Kechagias J.D. (2021). Sustainable Additive Manufacturing: Mechanical Response of Polyamide 12 over Multiple Recycling Processes. Materials.

[46] Anderson I. (2017). Mechanical Properties of Specimens 3D Printed with Virgin and Recycled Polylactic Acid. 3D Print. Addit. Manuf..

[47] Majgaonkar P., Hanich R., Malz F., Brüll R. (2021). Chemical Recycling of Post-Consumer PLA Waste for Sustainable Production of Ethyl Lactate. Chem. Eng. J..

[48] Hegedus T., Kreuter P., Kismarczi-Antalffy A.A., Demeter T., Banyai D., Vegh A., Geczi Z., Hermann P., Payer M., Zsembery A. (2022). User Experience and Sustainability of 3D Printing in Dentistry. Int. J. Environ. Res. Public Health.

[49] Nagata K., Muromachi K., Kouzai Y., Inaba K., Inoue E., Fuchigami K., Nihei T., Atsumi M., Kimoto K., Kawana H. (2023). Fit accuracy of resin crown on a dental model fabricated using fused deposition modeling 3D printing and a polylactic acid filament. J. Prosthodont. Res..

[50] Tagliaferri V., Trovalusci F., Guarino S., Venettacci S. (2019). Environmental and Economic Analysis of FDM, SLS and MJF Additive Manufacturing Technologies. Materials.

[51] Joda T., Zarone F., Ferrari M. (2017). The complete digital workflow in fixed prosthodontics: A systematic review. BMC Oral Health.

[52] Joda T., Brägger U. (2016). Time-efficiency analysis of the treatment with monolithic implant crowns in a digital workflow: A randomized controlled trial. Clin. Oral Implant. Res..

[53] Mühlemann S., Greter E.A., Park J.-M., Hämmerle C.H.F., Thoma D.S. (2018). Precision of digital implant models compared to conventional implant models for posterior single implant crowns: A within-subject comparison. Clin. Oral Implant. Res..

[54] Chiu A., Chen Y.-W., Hayashi J., Sadr A. (2020). Accuracy of CAD/CAM Digital Impressions with Different Intraoral Scanner Parameters. Sensors.

[55] Ren S., Jiang X., Lin Y., Di P. (2021). Crown Accuracy and Time Efficiency of Cement-Retained Implant-Supported Restorations in a Complete Digital Workflow: A Randomized Control Trial. J. Prosthodont..

[56] Al Hamad K.Q., Al-Rashdan R.B., Al-Rashdan B.A., Baba N.Z. (2021). Effect of Milling Protocols on Trueness and Precision of Ceramic Crowns. J. Prosthodont..

[57] Hanon M.M., Zsidai L., Ma Q. (2021). Accuracy investigation of 3D printed PLA with various process parameters and different colors. Mater. Today.

[58] Kamio T., Hayashi K., Onda T., Takaki T., Shibahara T., Yakushiji T., Shibui T., Kato H. (2018). Utilizing a low-cost desktop 3D printer to develop a “one-stop 3D printing lab” for oral and maxillofacial surgery and dentistry fields. 3D Print. Med..

[59] Chen L., He Y., Yang Y., Niu S., Ren H. (2016). The research status and development trend of additive manufacturing technology. Int. J. Adv. Manuf. Technol..

[60] George E., Liacouras P., Rybicki F.J., Mitsouras D. (2017). Measuring and Establishing the Accuracy and Reproducibility of 3D Printed Medical Models. Radiographics.

[61] Rybachuk M., Mauger C.A., Fiedler T., Öchsner A. (2017). Anisotropic mechanical properties of fused deposition modeled parts fabricated by using acrylonitrile butadiene styrene polymer. J. Polym. Eng..

[62] Mohamed O.A., Masood S.H., Bhowmik J.L. (2021). Modeling, analysis, and optimization of dimensional accuracy of FDM-fabricated parts using definitive screening design and deep learning feedforward artificial neural network. Adv. Manuf..

[63] Katsoulis J., Mericske-Stern R., Rotkina L., Zbären C., Enkling N., Blatz M.B. (2014). Precision of fit of implant-supported screw-retained 10-unit computer-aided-designed and computer-aided-manufactured frameworks made from zirconium dioxide and titanium: An in vitro study. Clin. Oral Implant. Res..

[64] Al-Meraikhi H., Yilmaz B., McGlumphy E., Brantley W., Johnston W.M. (2017). In vitro fit of CAD-CAM complete arch screw-retained titanium and zirconia implant prostheses fabricated on 4 implants. J. Prosthet. Dent..

[65] Yilmaz B., Kale E., Johnston W.M. (2018). Marginal discrepancy of CAD-CAM complete-arch fixed implant-supported frameworks. J. Prosthet. Dent..

[66] Presotto A.G.C., Bhering C.L.B., Mesquita M.F., Barão V.A.R. (2017). Marginal fit and photoelastic stress analysis of CAD-CAM and overcast 3-unit implant-supported frameworks. J. Prosthet. Dent..

[67] Yilmaz B., Alshahrani F.A., Kale E., Johnston W.M. (2018). Effect of feldspathic porcelain layering on the marginal fit of zirconia and titanium complete-arch fixed implant-supported frameworks. J. Prosthet. Dent..

[68] Molinero-Mourelle P., Cascos-Sanchez R., Yilmaz B., Lam W.Y.H., Pow E.H.N., Highsmith J.D.R., Gómez-Polo M. (2021). Effect of Fabrication Technique on the Microgap of CAD/CAM Cobalt–Chrome and Zirconia Abutments on a Conical Connection Implant: An In Vitro Study. Materials.

[69] Gedrimiene A., Adaskevicius R., Rutkunas V. (2019). Accuracy of digital and conventional dental implant impressions for fixed partial dentures: A comparative clinical study. J. Adv. Prosthodont..

[70] De La Cruz J.E., Funkenbusch P., Ercoli C., Moss M.E., Graser G.N., Tallents R.H. (2002). Verification jig for implant-supported prostheses: A comparison of standard impressions with verification jigs made of different materials. J. Prosthet. Dent..

[71] Krechmer J.E., Phillips B., Chaloux N., Shomberg R., Daube C., Manchanda G., Murray S., McCarthy A., Fonseca R., Thakkar J. (2021). Chemical Emissions from Cured and Uncured 3D-Printed Ventilator Patient Circuit Medical Parts. ACS Omega.

[72] Chan F.L., Hon C.-Y., Tarlo S.M., Rajaram N., House R. (2020). Emissions and health risks from the use of 3D printers in an occupational setting. J. Toxicol. Environ. Health A.

[73] Wojtyła S., Klama P., Baran T. (2017). Is 3D printing safe? Analysis of the thermal treatment of thermoplastics: ABS, PLA, PET, and nylon. J. Occup. Environ. Hyg..

[74] Dobrzyńska E., Kondej D., Kowalska J., Szewczyńska M. (2021). State of the art in additive manufacturing and its possible chemical and particle hazards—Review. Indoor Air.

[75] Qiu Q.Q., Sun W.Q., Connor J. (2017). Sterilization of Biomaterials of Synthetic and Biological Origin.

[76] Tipnis N.P., Burgess D.J. (2018). Sterilization of implantable polymer-based medical devices: A review. Int. J. Pharm..

[77] Ribeiro N., Soares G.C., Santos-Rosales V., Concheiro A., Alvarez-Lorenzo C., García-González C.A., Oliveira A.L. (2019). A new era for sterilization based on supercritical CO_2_ technology. J. Biomed. Mater. Res. Part B Appl. Biomater..

[78] Dai Z., Ronholm J., Tian Y., Sethi B., Cao X. (2016). Sterilization techniques for biodegradable scaffolds in tissue engineering applications. J. Tissue Eng..

[79] Davila S.P., Rodríguez L.G., Chiussi S., Serra J., González P. (2021). How to Sterilize Polylactic Acid Based Medical Devices?. Polymers.

